# Allergy Diagnosis in Children and Adults: *Performance of a New Point-of-Care Device, ImmunoCAP Rapid*

**DOI:** 10.1097/WOX.0b013e3181aed85c

**Published:** 2009-07-15

**Authors:** Gunilla Hedlin, Carmen Moreno, Carl Johan Petersson, Gunnar Lilja, Félix Lorente Toledano, Antonio Nieto García, Lennart Nordvall, Mona Palmqvist, Sabina Rak, Staffan Ahlstedt, Magnus P Borres

**Affiliations:** 1 Astrid Lindgren Children's Hospital, Karolinska University Hospital, Stockholm, Sweden; 2 Servicio Allergica-Adultos, Hospital Reina Sofia, Córdoba, Spain; 3 Sachs' Children's Hospital, Stockholm, Sweden; 4 Sección Alergia Pediátrica, Hospital Clínico-Universitario Salamanca, Salamanca, Spain; 5 Sección Alergia Pediátrica, Hospital La Fe-Materno Infantil, Valencia, Spain; 6 Uppsala University Children's Hospital, Uppsala, Sweden; 7 Department of Respiratory Medicine and Allergology, Sahlgrenska University Hospital, Göteborg, Sweden; 8 Phadia AB, Uppsala, Sweden; 9 Institute of Environmental Medicine, Karolinska Institute, Stockholm; 10 Department of Pediatrics, Sahlgrenska Academy at Göteborg University, Göteborg, Sweden

**Keywords:** IgE antibody allergy diagnosis, point-of-care test

## Abstract

**Background:**

Allergy is a serious problem affecting approximately 1 of 4 individuals. The symptoms with and without allergy etiology are often difficult to distinguish from each other without using an IgE antibody test. The aim of this study was to investigate the performance of a new point-of-care (POC) test for IgE antibodies to relevant allergens in Europe.

**Methods:**

IgE antibodies from children and adults with allergies recruited from allergy clinics in Sweden and Spain were analyzed for 10 allergens, suitable for the age groups, using the new POC test and ImmunoCAP laboratory test. The IgE antibody level best discriminating between positive and negative results (the cutoff point) for the different allergens of the POC test and the efficacy of the POC and the ImmunoCAP laboratory tests for diagnosing allergy compared with that of clinical diagnosis were investigated.

**Results:**

The estimated cutoffs for the different allergens in the POC test ranged from 0.70 to 2.56 kU_A_/L. Taking into account all positive allergen results in a given patient, the POC test could identify 95% of the patients with allergies. Seventy-eight percent of the allergen-specific physicians' diagnoses were identified and 97% of the negative ones. Most allergens exhibited good performance, identifying about 80% of clinically relevant cases. However, dog, mugwort, and wall pellitory would benefit from improvement.

**Conclusions:**

The POC test will be a valuable adjunct in the identification or exclusion of patients with allergies and their most likely offending allergens, both in specialist and general care settings.

## 

The prevalence of allergic diseases has been increasing during the past several decades, both in the Western world and in developing countries where a more "Westernized" lifestyle has been blamed [1-3]. The diagnosis of allergic diseases demands confirmation of specific IgE antibodies in patients with symptoms. This has traditionally been done using skin prick tests (SPT) that give immediate results, but are difficult to standardize for inexperienced users. Specific IgE antibodies have also been demonstrated for more than 30 years in the blood by using fairly advanced and time-consuming laboratory tests [4]. Thus, there is a need for a well-standardized, simple, and quick point-of-care (POC) test that can be easily performed in the physician's office. The following 4 reasons why a POC test for specific IgE is needed were identified. First, allergy may affect one fourth of the population in the Western world [1,2]. These patients are seen by both allergists, who have good experience in allergy diagnosis, and by pediatricians and family physicians who have less experience in this field.

Second, only 1 to 2 of 3 patients suffering from such symptoms may actually be allergic [5,6]. It is frequently difficult to distinguish between the symptoms of an allergic explanation from a nonallergic one (wheezing, shortness of breath, dyspnea, bronchi, cough, and chest tightness; nasal congestion, sneezing, rhinorrhea, itching of nose, ears, and eyes, and postnasal drainage; nausea, vomiting, reflux, constipation, abdominal cramping, and diarrhea; itchy, erythematous, and scaly skin). The identification of allergy in patients is considerably hampered without access to an IgE antibody test [7-11].

Third, there is a consensus that knowledge of the allergic status and understanding of the environmental context of a patient will allow a more adequate choice of therapy and efficient management of the symptoms and evolving disease [12,13]. Likewise, it is important to be able to exclude allergy from other reasons for the symptoms. In particular, the presence of allergy and elevated IgE antibody levels represents a risk for acute and complicating reactions over time [14].

Fourth, even if there are hundreds of substances that can be allergenic, practical clinical experience demonstrates that a limited number of the most common allergens in the environment will identify more than 90% of the individuals with allergies [15].

The most effective clinical tool should be simple enough to be used in the physician's office and possess the ability to verify or exclude the presence of allergy. In addition, this tool should also not be confused with low-grade sensitization that can be difficult to interpret,[4,14,16-18] even in combination with case history and physical examination [7,10,11,14,19].

We report on the evaluation of a safe and simple tool for POC testing of relevant IgE antibodies to environmental allergens in Europe that gives results in a few minutes.

Whereas Diaz-Vazquez et al and Eigenmann et al [20,21] evaluated this POC exclusively in children, this study also addressed adults with similar symptoms.

## Materials and Methods

### Patients

This one-visit multicenter study involved 229 patients at 7 allergy centers. In Sweden, 3 centers were pediatric clinics and 1 center an adult clinic. In Spain, 2 centers were pediatric clinics and 1 was an adult clinic. Patients with a history of symptoms and sensitization to 1 or more of the allergens to be evaluated were invited to participate in the study. Patients between 0 and 18 years of age were recruited in the pediatric clinics and patients between 19 and 65 years of age in the adult clinics. For inclusion, the patient needed to have ongoing symptoms of wheeze/asthma and/or rhinitis. Also, the patient had to agree to have a 110-*μ*L capillary and a 4-mL venous blood sample taken and to accept participation by signing an informed consent form. Subjects infected with HIV and those with a history of hepatitis were excluded from the study. Case history of allergy-like symptoms and potential factors giving the patients symptoms of allergy were recorded. The distribution of patients by demographic characteristics and participating country was similar for both pediatric and adult clinics and can be provided by the authors upon request. In the 2 adult clinics, there was a predominance of females, particularly in Sweden. The adult patients in Spain were somewhat younger, median 26 years compared with 36 years in Sweden.

The protocol for this study was reviewed and approved by the following: Comite Etico Investigacion Clinica Hospital "Reina Sofia", Cordoba, Spain; Comite Etico Investigacion Clinica Hospital Universitario La Fe, Valencia, Spain; Comite Etico Investigacion Clinica Hospital Universitario Salamanca, Spain; Forskningsetikkommittén vid Karolinska Institutet, Stockhlm, Sweden; and Forskning-setikkommittén vid Göteborgs Universitet, Gothenburg, Sweden. Patient informed-consent forms were signed by all patients--parents and children (age permitting) and adults--before enrollment.

### Point-of-Care Test

Specific IgE antibodies for a total of 10 allergens were qualitatively determined using 2 test profiles representative of the regions from where the patients were recruited: 1 for children (ImmunoCAP Rapid Wheeze-Rhinitis Child) and 1 for adults (ImmunoCAP Rapid Asthma/Rhinitis Adult) (Phadia AB, Uppsala, Sweden). Each profile included 2 control areas and 10 allergens identifiable on an individualized basis. For the 2 profiles there were 8 common and 2 profile-specific allergens. The common allergens were timothy (*Phleum pratense*), birch (*Betula verrucosa*), olive (*Olea europaea*), wall pellitory (*Parietaria judaica*), mugwort (*Artemisia vulgaris*), cat dander, dog dander, and house dust mite (*Dermatophagoides pteronyssinus*). The 2 profile-specific allergens for the child test were egg white and milk and, for the adult test, *Alternaria alternata *and cockroach (*Blatella germanica*). The compositions of the profiles were based on the most frequent specific allergens known to occur (in Europe) in the 2 target populations: children and adults with allergy-related symptoms. The allergen reagents on the POC test included natural extracts and purified allergen components and recombinant allergens. To achieve an optimal allergen preparation, a combination of purified original extract spiked with critical allergen components was often necessary. Examples of preparations where an increased sensitivity without a decrease in specificity could be achieved were for olive pollen, where the original extract was spiked with purified Ole e 1 (a major component in olive pollen) and for dog spiked with recombinant Can f 1 and Can f 2 (major components in dog epithelia).

At each center, the POC test was performed by an appointed study nurse and the results remained blind for the physician until the end of the study. The total time for running the POC test was 20 minutes after 110 *μ*L of blood was applied to the assay device. Plasma separated from blood cells by capillary force and flowed onto the 2 parallel visible test strips. If present, specific IgE antibodies bound to the zone on the test strip containing the corresponding allergen. After 5 minutes, a developer solution was added into a separate well, releasing a dried gold conjugate that similarly flowed onto the test trips. After an additional 15 minutes, the conjugate formed a visible pink-red complex with any bound IgE antibodies for a positive allergen in the test strip. A invisible line was read as negative, indicating that specific IgE antibodies were undetectable with this POC test. Two control windows, one on each strip, indicated whether the test run should be approved or not. The principle of the test has also been published elsewhere [20,22].

Reproducibility studies performed by the manufacturer have shown the same response (positive or negative) in 94% of the tests when repeated on different occasions and across different batches. High levels of total IgE, up to 3000 kU/L, did not interfere with the test results. Similarly, no measurable cross-reactivity was observed of the IgE-specific conjugate with human IgG, IgA, IgD, or IgM and no interference from hemoglobin, bilirubin, triglycerides, or cholesterol was detected within normal concentration ranges. Test results were not affected by hematocrit levels up to 48% [22].

### Quantitative Allergen-Specific IgE Measurements

For all patients the quantitative specific IgE antibody level was determined for each allergen included in the new tests by using the venous blood sample (ImmunoCAP, Phadia AB, Uppsala, Sweden). A patient with IgE antibody levels above 0.35 kU_A_/L to an individual allergen was considered sensitized.

### Clinical Evaluation of the POC Test

Each patient was examined separately for each of the 10 allergens where each allergen was classified as "positive", "negative", or "inconclusive". At each center, there was 1 physician responsible for the clinical judgment of the participating patients. The physician's judgment was based on, besides case history and physical examination, SPT results (SPT cutoff point: wheal diameter ≥ 3 mm) and/or Immuno-CAP specific IgE determinations (cutoff point: ≥ 0.35 kU_A_/L) when necessary. An inconclusive diagnosis was set when case history and the laboratory IgE test result or any previous test result did not correspond.

For the evaluation of the diagnostic performance of the POC test, the results for each allergen were compared with the positive and negative classifications made by the physicians.

### Data Analysis and Statistical Methods

The percentage agreement of a positive POC test with the physician's positive diagnosis and the percentage agreement of a negative POC test with the physician's negative diagnosis were calculated for each separate allergen and for all allergens collectively.

For each allergen, receiver-operating characteristic (ROC) analysis [23] was used in a reversed manner to estimate cutoff levels for the quantitative laboratory test, using the results of the POC test as the reference, that is, to estimate specific IgE levels that best discriminated between positive and negative results of the POC test. This was done using all available observations, irrespective of allergen diagnosis.

On the basis of the physician's positive or negative classification, the clinical performance of the POC test was compared with the performance of the ImmunoCAP Specific IgE using both the standard cutoff and the cutoff estimated from the data.

Statistical analyses were performed using the SAS statistical software system (v8.2). Microsoft Excel 2003 was used for figures and plots.

## Results

Overall, in the pediatric clinics, 73 children (60%) presented with a diagnosis of asthma and 101 children (84%) with rhinitis. The prevalence of asthma and allergic rhinitis in Swedish children was 90% for each, whereas that in the children from Spain was 24% for asthma and 76% for rhinitis. In the adult clinics, a similar number of patients were seen in the 2 countries with 90% symptoms for rhinitis and 64% for asthma. In the pediatric clinics, the most common self-reported allergens were tree pollen (55%), cat dander (50%), and grass pollen (49%). Infection headed the list of nonallergen triggering factors (49%). The percentage of each triggering factor was generally higher in Sweden than in Spain. In the adult clinics, the most common allergens reported were tree pollen (75%), house dust mite (62%), and cat dander (46%). Nonallergen triggering factors, such as smoking (57%), infection (51%), and exercise (48%), were similar in both countries.

### Estimated Cutoffs per Allergen for the POC Test Calculated Using the Quantitative Laboratory Blood Test

The cutoff for each allergen of the new POC test was calculated with the laboratory test by estimating the kU_A_/L levels giving the best discrimination between positive and negative POC test results. Different levels were found for the different allergens, ranging from a low of 0.70 kU_A_/L for milk to a high of 2.56 kU_A_/L for wall pellitory (Table 1). This gave a percentage agreement between the laboratory test results with the positive and negative POC test results ranging from 92% and 94% respectively for milk to 77% and 95% respectively for wall pellitory, with all other allergens falling in between.

**Table 1:** Agreement of the POC and Laboratory Test (Using Different Cutoff Values) With Doctor's Diagnosis

			Doctor's Positive Allergen-Specific Diagnoses				Doctor's Negative Allergen-Specific Diagnoses		
					
Allergen	Estimated POC Cutoff (kU_A_/L)	n	Pos. POC (%)	sIgE ≥ 0.35 kU_A_/L (%)	sIgE ≥ POC Cutoff (%)	n	Neg. POC (%)	sIgE < 0.35 kU_A_/L (%)	sIgE < POC Cutoff (%)
House dust mite	0.82	80	82	99	95	117	93	93	97
Cat	0.72	98	81	96	87	101	98	97	100
Dog	1.81	74	51	97	68	103	100	79	99
Timothy grass	2.42	125	82	98	84	67	97	94	100
Birch	2.15	81	90	100	89	93	95	82	100
Olive	1.41	63	81	100	84	86	100	86	99
Mugwort	1.27	32	53	94	72	128	95	88	98
Wall pellitory	2.56	17	82	100	71	133	97	84	99
Egg white	0.90	32	84	94	84	86	97	98	99
Cow's milk	0.70	25	84	100	92	89	98	94	99
*A. alternata*	2.33	24	88	96	92	74	97	97	100
Cockroach	--	2	50	100	--	87	99	97	--

### Performance of the POC Test and the Laboratory Test Compared with the Physician's Diagnosis

In total, 218 of the 229 patients tested positive to at least 1 allergen in the POC test. Of these patients, 216 had at least 1 positive allergen diagnosis made by the physician. In addition, 9 of the 11 subjects with negative POC test had at least 1 such positive allergen diagnosis made by the physician. Thus, of the 225 patients with at least 1 positive clinical allergen diagnosis, the POC test recognized 216 or 95% of them.

**Figure 1:**
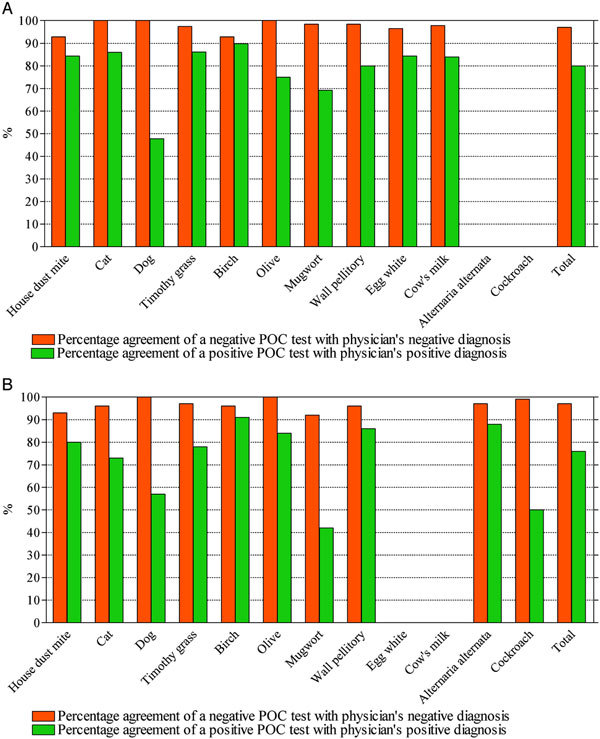
**Percentage agreement of the POC test results with the physician's diagnosis for (A) children and (B) adults**.

In total, 2284 allergen-specific diagnoses were made in the 229 patients. The number of positive and negative diagnoses were 653 and 1165, respectively. The number of inconclusive diagnoses was 466. For each single allergen the distribution of the POC test results versus the physicians' positive and negative diagnoses is shown in Table 1. In total, 78% of the physicians' positive allergen diagnoses were associated with a positive result in the POC test. In the case of the physicians' negative allergen diagnoses, the POC test results were negative in 97% of the cases. This resulted in an overall agreement of the study test compared with the positive and negative diagnoses of 90%. The results from children and adults were comparable (Figure 1) as were those from Spain and Sweden (not shown), justifying analysis as a single group.

The performance of the POC test compared with that of the laboratory test using the clinical diagnosis as the discriminator is shown in Figure 1 and Table 1. The agreement of a positive POC test result and a laboratory test result above the cutoff value, as estimated for the individual allergens in the POC test with a clinical diagnosis, was above 80% for most allergens (Table 1). However, the concordance of the results was lower for dog and mugwort. The concordance of positive POC test results with results of positive clinical diagnosis was compared with the concordance of positive specific IgE results from the laboratory test using the ordinary 0.35 kU_A_/L cutoff for the individual allergens with results of positive clinical diagnosis. It was found that the performance of the POC test was about 15% lower, with the exception of dog and mugwort where the differences were 46% and 41% lower, respectively (Table 1).

The corresponding analysis of the agreement of negative POC test results with negative clinical diagnosis showed concordant results, with values well above 90% and, for many allergens, close to 100% when compared with the laboratory test (Table 1). As expected, when compared with the regular 0.35 kU_A_/L cutoff, most allergens showed better agreement with the physician's diagnosis in the POC test, albeit in most instances less than 15% than for the laboratory ImmunoCAP test. The exception was for dog where there was a 21% better outcome in the POC test. In all these analyses the results were very similar for both children and adults (Figure 1) and both Spain and Sweden (not shown).

In the recruited patients, the number of inconclusive clinical classifications per allergen was, in total, 466 (20%), that is, discordant results between clinical history and laboratory IgE antibody test, or any previous test results. Of these results, 30% were positive in the POC test. One of the most difficult allergens to diagnose in this study, resulting in inconclusive diagnosis, was wall pellitory in both countries (31% in Spain and 18% in Sweden). Likewise, inconclusive diagnosis was high for birch in Spain (34%) and for olive tree in Sweden (25%).

## Discussion

The POC test was able to identify 95% of the patients having a diagnosis of allergic disease. Despite this excellent result this new test exhibited somewhat lower sensitivity than the conventional laboratory test ImmunoCAP [24]. It could correctly identify 78% of the allergen-specific diagnoses and 97% of the negative allergen-specific diagnoses, giving an overall concordance of 90%. These results are in accordance with the results obtained in previous studies using the POC test [20,21]. However, in this study it was found that the results between the specific allergens varied within a wide range; that is, the range in sensitivity was from around 50% for dog, mugwort, and cockroach to around 90% for *A. alternata *and birch. Concerning the relatively low values for the allergens for dog, mugwort, and cockroach, they would probably benefit from a manufacturer's test improvement.

To increase the prevalence of clinical allergy and sensitization to the employed allergens, it was decided to include patients previously recognized as having allergies. This approach enriched the population of people with allergies compared with a consecutive enrollment but still allowed analysis of clinical and serological reactivity to both offending and nonoffending allergens. Therefore, it was not surprising that 218 patients of 229 were positive in the POC test to 1 or more of the employed allergens and 225 patients had a positive general allergy diagnosis. In an attempt to cover different regions of exposure, patients from both Spain and Sweden were included, consisting of both adults and children. There was no indication that the inclusion of patients from northern and southern Europe and of different ages blurred the results. Individual allergen results showed that there was a difference in the number of inconclusive results in patients for birch pollen in Spain and for olive and wall pellitory in Sweden. This may be due to the fact that these pollens do not occur in the respective region and the results may be due to cross-reactivity between allergens. For example, ash (Fraxinus), lilac (Syringa), and privet (Ligustrum) are trees present in the northern European region and belong to the same family (Oleaceae) as olive (Olea). As such, these results may be considered as irrelevant.

In the present study there was a positive or negative diagnosis for the majority of the individual allergens in the recruited patients. However, there were also those with inconclusive diagnoses. Many of those cases exhibited low IgE antibody levels to the allergens (unpublished results). This finding may be taken as proof of the difficulty to link low-grade sensitization and low IgE antibody levels with actual clinical symptoms.

The POC test exhibited somewhat lower sensitivity than the laboratory test. Therefore, the cutoffs for the different allergens in the POC test were estimated and interpreted in relation to the laboratory test using a reversed ROC method. Normally, ROC analysis could be used to select a cutoff for a quantitative test versus a qualitative (positive or negative) reference. In this study, however, the laboratory test was used as the quantitative test and the test in question, the POC test, as the qualitative reference. The estimation of the cutoff could thus be performed to provide the best agreement with the laboratory test ImmunoCAP Specific IgE. Many individuals may have detectable IgE antibodies without having any symptoms at the given moment. This is seen in both small children [25] and adults [26] who become sensitized before symptoms evolve. Furthermore, in food allergy, probability curves have been developed to find a better association between clinical reactivity and IgE antibody levels higher than the generally accepted cutoff level of 0.35 kU_A_/L [27-30]. Similarly, in respiratory allergy, elevated IgE antibody levels have been shown to be more closely associated with current symptoms [10,14,18,19] and decreased lung function [18] than lower IgE antibody levels. This pattern is also accentuated in determining the risk for acute asthma exacerbations [14]. After this, one can speculate that the use of a test with a higher cutoff will probably identify patients with a higher likelihood of allergy problems and could therefore be appropriate as a first-line diagnostic tool, especially if used in settings with patients presenting with a lower prevalence of allergy. Furthermore, it can be seen in the literature that different authors choose cutoffs above 0.35 kU_A_/L in their clinical practical use of IgE antibody testing [31].

There was a concern that patients seen by a specialist are generally more heavily sensitized with higher IgE antibody levels than patients seen by primary care physicians. This may lower the usefulness of a less sensitive test such as the POC test compared with the regular laboratory test. However, with re-analysis of previously published data from primary care in Italy and Spain [9] and in allergist clinics in Italy, Germany, The Netherlands, and England,[32] we were able to confirm that this was not the case (unpublished results).

## Conclusions

In conclusion, this study revealed that this POC test for IgE antibodies has a high capability of identifying patients with allergic etiology for their symptoms, despite somewhat higher cutoffs for the different allergens employed than those currently used in laboratory tests.
